# Supplementary figures and images for: Upregulation of CD1d and ULBP3 on B cells from healthy donors and chronic lymphocytic leukaemia patients does not prime them for killing by γδ T cells
Source: PLoS One. 2026 Apr 20;21(4):e0346684. doi: 10.1371/journal.pone.0346684 (PMC13095099; doi:10.1371/journal.pone.0346684)

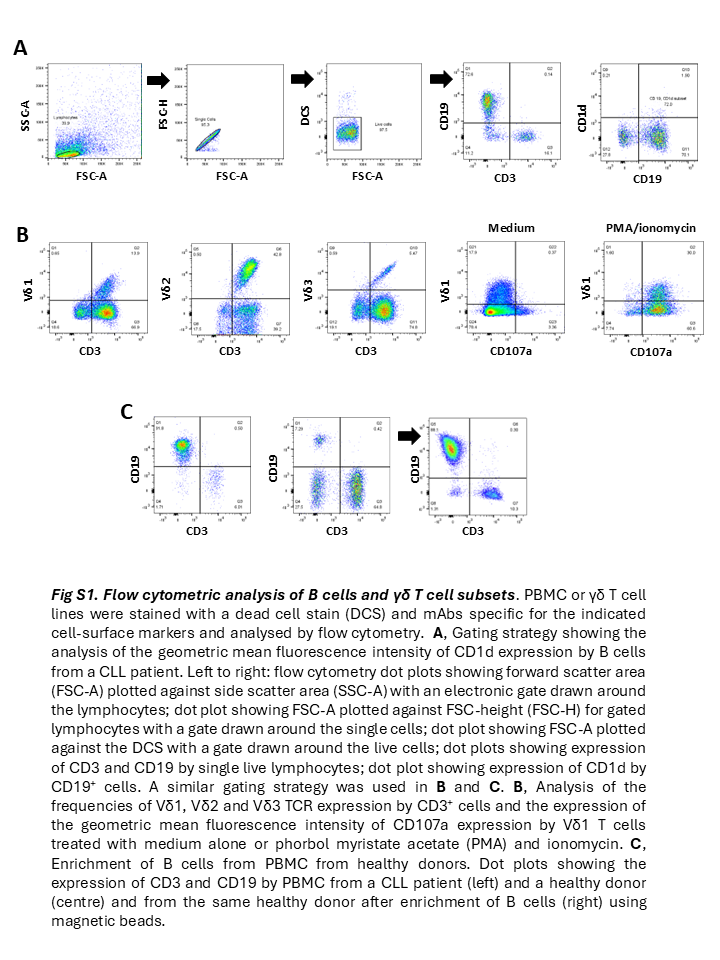

Supplement: S1 Fig — (TIF) [file pone.0346684.s001.tif]

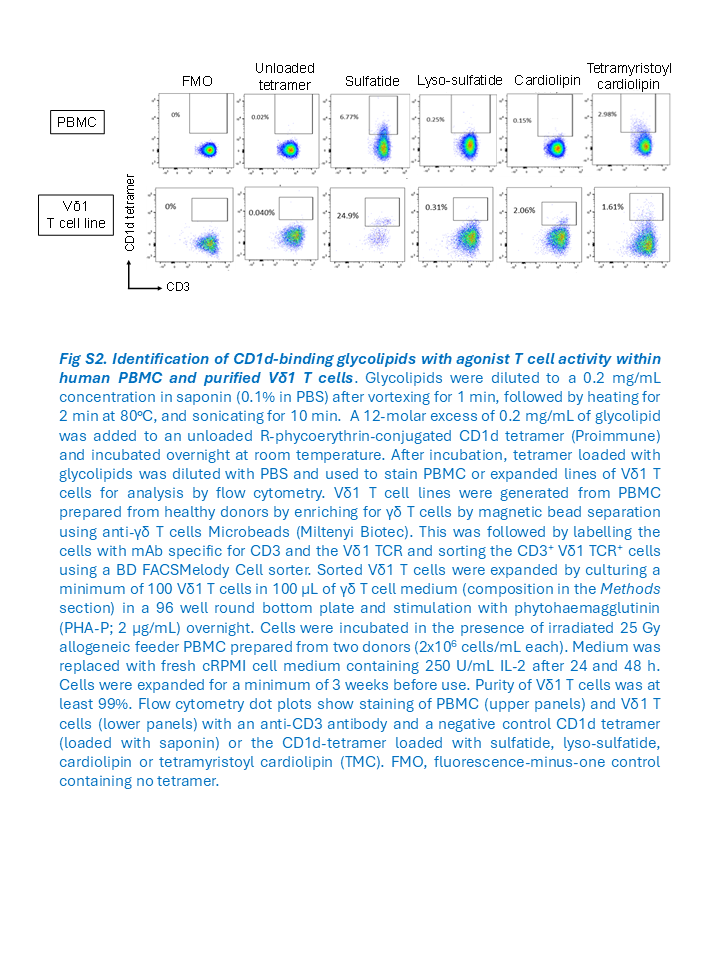

Supplement: S2 Fig — (TIF) [file pone.0346684.s002.tif]
