# Supplementary material for: Upregulation of CD1d and ULBP3 on B cells from healthy donors and chronic lymphocytic leukaemia patients does not prime them for killing by γδ T cells
Source: PLoS One. 2026 Apr 20;21(4):e0346684. doi: 10.1371/journal.pone.0346684 (PMC13095099; doi:10.1371/journal.pone.0346684)
Supplement: Attachment — Submitted filename: Response to Reviewers.docx [file pone.0346684.s005.docx]

**Responses to editor’s and reviewers’ comments on manuscript PONE-D-25-60204**

‘Upregulation of CD1d and ULBP3 on B cells from healthy donors and chronic lymphocytic leukaemia patients does not prime them for killing by γδ T cells’ By David et al.

**Comments from Editor**

**Comment 1:** Please ensure that your manuscript meets PLOS ONE's style requirements, including those for file naming. The PLOS ONE style templates can be found at

<https://journals.plos.org/plosone/s/file?id=wjVg/PLOSOne_formatting_sample_main_body.pdf> and

<https://journals.plos.org/plosone/s/file?id=ba62/PLOSOne_formatting_sample_title_authors_affiliations.pdf>

**Response:** We have prepared the manuscript text and the titles/authors/affiliations sections according to the PLOS ONE style templates. The figures and tables are mentioned consecutively in the text and arranged as requested. The file names have been changed in accordance with those stated in the PLOS ONE guidelines. We have updated the manuscript to reflect these changes as follows in the uploaded figures:

- Changed all Level 1 headings (abstract/intro/methods/results/discussion) to 18 pt font.
- Changed all Level 2 subheadings to 16 pt font.
- Changed figure references in main text to ‘Fig’ or ‘Figs’.
- Changed file names to ‘Response to Reviewers’, ‘Revised Article with Changes Highlighted’ and ‘Manuscript’, as requested.
- Moved ‘Supporting information’ to after ‘References’ section (Line 730-734).
- Deleted superfluous ‘Funding information’, ‘Author Contributions’, ‘Data availability’ and extra ‘Ethical statement’ sections from the manuscript.
- Within-figure text has been changed to ‘Arial’ font.
- Each figure has been separated into individual files and converted/uploaded in the requested tiff format and named appropriately ‘Fig1.tif’, etc.
- Figure captions moved to main text and removed from figure files.
- Figure dimensions altered to be within maximum dimensions of 7.5 inches wide by 8.75 inches tall.
- Additional figures have been added, and all figures have been re-ordered.
- Table 1 has been updated to include the samples utilised in the additional experiments that were undertaken as part of this revision. An additional three CLL patient-derived samples were used, and this has been included in the updated version of Table 1 (Lines 253-254), and throughout the text, as required. The updated table meets the PLOS ONE guidelines.
- Additional references have been added to support results from additional experiments (Line 707-715 and 722-723).

**Comment 2:** Please note that funding information should not appear in any section or other areas of your manuscript. We will only publish funding information present in the Funding Statement section of the online submission form. Please remove any funding-related text from the manuscript.

**Response:** The funding information section has been removed from the manuscript.

**Comment 3:** Your ethics statement should only appear in the Methods section of your manuscript. If your ethics statement is written in any section besides the Methods, please delete it from any other section.

**Response:** The ethics statement has been removed from the end of the manuscript, and now only belongs in the Methods section.

**Comment 4:** Please upload a new copy of Figures 1, 2, 3, 4, 5, 6, 7; and Supplementary Figure 1 as the detail is not clear. Please follow the link for more information:

<https://journals.plos.org/plosone/s/figures>

**Response:** A new copy of all figures has individually been uploaded in accordance with the PLOS ONE guidelines.

**Comment 5:** We note that there is identifying data in the Supporting Information file <David et al. Raw data.xlsx>. Due to the inclusion of these potentially identifying data, we have removed this file from your file inventory. Prior to sharing human research participant data, authors should consult with an ethics committee to ensure data are shared in accordance with participant consent and all applicable local laws.

Data sharing should never compromise participant privacy. It is therefore not appropriate to publicly share personally identifiable data on human research participants. The following are examples of data that should not be shared:

-Name, initials, physical address

-Ages more specific than whole numbers

-Internet protocol (IP) address

-Specific dates (birth dates, death dates, examination dates, etc.)

-Contact information such as phone number or email address

-Location data

-ID numbers that seem specific (long numbers, include initials, titled “Hospital ID”) rather than random (small numbers in numerical order)

Data that are not directly identifying may also be inappropriate to share, as in combination they can become identifying. For example, data collected from a small group of participants, vulnerable populations, or private groups should not be shared if they involve indirect identifiers (such as sex, ethnicity, location, etc.) that may risk the identification of study participants.

Additional guidance on preparing raw data for publication can be found in our Data Policy (<https://journals.plos.org/plosone/s/data-availability#loc-human-research-participant-data-and-other-sensitive-data>) and in the following article: <http://www.bmj.com/content/340/bmj.c181.long>.

Please remove or anonymize all personal information (<specific identifying information in file to be removed>), ensure that the data shared are in accordance with participant consent, and re-upload a fully anonymized data set. Please note that spreadsheet columns with personal information must be removed and not hidden as all hidden columns will appear in the published file.

**Response:** No identifying information could be found in the <David et al. Raw data.xlsx> file by authors. Data associated with patient samples has been labelled with fully anonymised labels such as ‘Donor 1, Donor 2, etc.’.

**Comment 6:** If the reviewer comments include a recommendation to cite specific previously published works, please review and evaluate these publications to determine whether they are relevant and should be cited. There is no requirement to cite these works unless the editor has indicated otherwise.

**Response:** Neither reviewer has recommended that any particular previously published article be referenced in the manuscript.

**Comments from reviewer #1 (Jing Ju):**

**Comment 1:** The cytotoxicity assays depend almost exclusively on CD107a surface expression as a surrogate for γδ T-cell degranulation. Although CD107a is a widely used marker, it does not always correlate with actual target-cell death, particularly in cases where immunological synapse formation or perforin-mediated killing is impaired. Incorporating direct cytotoxicity measurements—such as flow-based live/dead killing assays, or real-time imaging—across multiple effector-to-target ratios and time points would provide more definitive evidence regarding whether B cells are truly resistant to γδ T-cell–mediated killing.

**Response:** We agree with the reviewer’s comment that CD107a surface expression and therefore degranulation of γδ T cells does not necessarily correlate with target cell death alone. The reviewer makes an excellent point that this may be due to immunological synapse impairment, which is a common feature observed between dysfunctional conventional T cells and CLL cells *in vivo*.

To address this issue, further cytotoxicity assays were carried out as suggested. Firstly, a flow-based live/dead assay was conducted in co-cultures of expanded γδ T cell lines with patient-derived CLL cells, in which viability of CLL cells at various E:T ratios (1:1, 3:1, 6:1) was measured by propidium iodide uptake. These data can be seen in Figure 4 of the revised figures/manuscript. Interestingly, the viability of CLL cells did decrease upon co-culture, and this decrease was more pronounced at larger E:T ratios. This decrease in viability may reflect either non-degranulating cytotoxic mechanisms, cytotoxicity mediated by γδ T cells other than Vδ1, Vδ2 and Vδ3 T cells, or may be due to the highly stressful co-culture conditions, such as nutrient deficiency, local hypoxia, lactate accumulation or the harsh conditions reflected in physically crowded co-culture conditions. CLL cells are metabolically fragile and notoriously sensitive to oxidative stress and changes in pH and nutrient availability, all of which are heavily impacted by co-culture conditions with high E:T ratios. To clarify, the purpose of this study is to evaluate CD1d-mediated killing of CLL cells by γδ T cells, not killing in general. Given that ATRA significantly upregulates both CD1d and ULBP3 on CLL cells, and given the viability of CLL cells did not change between ATRA-treated and -untreated cells at any E:T ratios, this supports our claim that upregulation of CD1d and ULBP3 on B cells from healthy donors and chronic lymphocytic leukaemia patients does not prime them for killing by γδ T cells. To reflect this additional experiment, the following sections have been added to the main body of the updated manuscript:

- Lines 38 and 41-42 in the abstract
- Lines 156-157 in the updated methods
- Lines 324-333 in the updated results section
- New Figure 4
- Lines 474-489 in the updated discussion.

In addition, we investigated production of cytokines IFN-γ and TNF-α, two key inflammatory mediators of the anti-tumour response by γδ T cell subsets, in response to co-culture with patient-derived CLL cells treated with or without ATRA. In agreement with our previous CD107a-based assays, no difference in IFN-γ or TNF-α production was found when expanded γδ T cells were co-cultured with CLL cells. These data can be seen in a new Figure 6 (B) of the revised manuscript. The relevant changes are as follows:

- Line 43 in the abstract
- Lines 147-148 in the Methods
- Lines 379-384 in the Results
- New Figure 6B
- Lines 393-399 in the updated Fig 6 caption
- Lines 450 in the updated discussion.

Overall, our data show that expanded γδ T cells 1) lack CD107a externalisation and therefore do not degranulate and 2) do not produce two key anti-tumour cytokines in response to CLL cells. These two critical functional readouts suggest that γδ T cells do not actively target CLL cells for killing.

**Comment 2:** All functional assays rely on long-term expanded γδ T-cell lines derived from healthy donors. Such expanded lines may exhibit altered receptor expression or activation thresholds, and allogeneic interactions do not accurately model autologous physiology. Including freshly isolated T cells and ideally autologous γδ T cells from the same individuals would help exclude artifacts arising from in vitro expansion or allogeneic mismatch and strengthen confidence in the negative findings.

**Response:**

We acknowledge the utility of using fresh, unexpanded T cell populations from CLL patients, and how this may differ from using expanded γδ T cells from healthy donors. We used expanded γδ T cells in order to uphold translational relevance, since all current clinical trials investigating γδ T cells in cancer, use long-term expanded γδ T cell lines. Additionally, as PBMC samples obtained from CLL patients are generally at least 90% CLL cells, γδ T cells numbers would be so low that any functional killing assays would be extremely difficult to carry out. To overcome this problem, we used buffy coat packs obtained from the Irish Blood Transfusion Service as the sources of γδ T cells. The use of allogeneic effector cells is also in line with expectations that γδ T cells can be used as ‘off-the-shelf’ cellular therapies, with one donor providing for multiple recipients. We greatly appreciate the reviewer’s insightful suggestion but believe that the use of freshly-isolated γδ T cells is beyond the scope of this study and better suited for future work. We have, however, examined if the expanded γδ T cells express exhaustion markers and this extra experiment is described below in the response to Comment 3.

**Comment 3:** RAR-α agonist treatment may induce additional immunomodulatory molecules on B cells beyond CD1d and ULBP3. Assessing inhibitory ligands such as PD-L1, or other immunosuppressive markers would clarify whether these pathways contribute to the lack of γδ activation. Furthermore, the study does not evaluate whether RAR-α agonists directly alter γδ T-cell viability, metabolic state, or cytokine production/proliferation. These controls are important to exclude unintended suppression or modulation of γδ T-cell function by the compounds.

**Response:** We agree with this reviewer that evaluation of expression of inhibitory ligands on CLL cells and their receptors on our γδ T cell lines would elucidate whether functional exhaustion may contribute to the lack of γδ activation in response to CLL cells. While we are unable to obtain repeat samples of CLL cells to examine PD-L1 expression, we have generated 3 new lines of γδ T cells and measured the surface expression of four key inhibitory molecules - PD-1, CTLA-4, TIM-2, and LAG-3 - on Vδ1, Vδ2 and Vδ3 T cells at day 24 in culture, directly before use in cytotoxicity assays. These data can be seen in Figure 3 (B) of the revised figures/manuscript. High expression of PD-1 and CTLA-4 was observed on Vδ2 and Vδ3 T cells from two γδ T cell lines (GDTC 1, GDTC 2). Moderate expression of TIM-3 was observed on Vδ2 and Vδ3 T cells from two γδ T cell lines (GDTC 1, GDTC 2). LAG-3 was lowly expressed on all three lines. We sincerely thank the reviewer for this suggestion, which we believe has strengthened the study. To reflect this additional experiment, the following sections have been added to the main body of the updated manuscript:

- Line 148 in the Methods
- Lines 299-306 in the Results
- New Figure 3B
- Lines 318-320 in the updated Fig 3 caption
- Lines 540-543 in the updated discussion.

The effect of RAR-α agonists on γδ T cell mediated cytotoxicity or viability is not relevant in this study as the γδ T cells were not themselves exposed to RAR-α agonists. CLL cells were pre-treated with RAR-α agonists for up to 72 hr and then washed and added to equal proportions of γδ T cell lines. Viability of γδ T cell lines was not altered by co-culturing them with CLL cells, with similar viabilities seen in γδ T cells cultured alone (data not shown). We apologise if this was not made clear in the Methods section.

**Comment 4:** Additional functional profiling would provide a more complete understanding of γδ T-cell activation status. Quantifying granzyme B, perforin, IFN-γ, TNFα, and activation markers such as CD69 or CD25 would help determine whether γδ T cells exhibit partial, non-cytolytic, or exhausted activation states not reflected by CD107a alone.

**Response:** We strongly agree with the reviewer that further functional profiling is essential to elucidate whether γδ T cells actively respond to CLL cells. As recommended, we have performed an additional experiment using 3 new γδ T cell lines, quantifying IFN-γ and TNF-α producing Vδ1, Vδ2 and Vδ3 T cells following co-culture with CLL cells treated with or without ATRA. While all γδ T cell subsets in all 3 lines produced these cytokines in response to PMA and ionomycin treatment, no IFN-γ or TNF-α production, and therefore no non-cytolytic functionality, was observed in response to CLL cells. These data can be seen in Figure 6 (B) of the revised figures/manuscript. In addition to this, CD69 expression on γδ T cell lines was assessed and this did not change in response to CLL cells (data not shown). We sincerely thank the reviewer for this suggestion, which we believe has strengthened the finding that γδ T cells do not actively target CLL cells in this study. Additional text in the revised manuscript describing this experiment can be found in the following locations:

- Line 43 in the abstract
- Lines 147-148 in the Methods
- Lines 379-384 in the Results
- New Figure 6B
- Lines 393-399 in the updated Fig 6 caption
- Lines 450 in the updated discussion.

**Comments from Reviewer 2 (anonymous reviewer)**

**Comment 1 (abstract):** Authors claim that “This study reveals that human Vδ1 T cell cytotoxicity against CLL B cells may be disease stage dependent and requires B cell priming and selective expansion and activation of specific γδ T cell subsets.” However, no results or discussion are presented regarding how cytotoxicity varies with disease stage.

**Response:** We agree with the reviewer that no results or discussion are presented in the original manuscript bearing fact to this claim. We apologise and have updated the abstract to say ‘’This study *suggests* that…’’ We have also revised the discussion to include reference to a recent paper that reports that expanded γδ T cells display reduced cytotoxic potential against increasing CLL cell burden. We thank the reviewer for making this clarification.

- Line 45 in the abstract
- Lines 528-530 in the updated discussion.

**Comment 2 (introduction):** The introduction is clear and provides sufficient background to set the context and the relevance of this study.

**Response:** We appreciate the reviewer’s kind comments.

**Comment 3 (methods):** The authors report using an E:T ratio of 1:1 in their co-cultures. While this is acceptable, γδ T-cell-mediated responses often require higher ratios (e.g., 5:1 or 10:1) to detect degranulation and target killing, particularly against primary B cells.

**Response:** We agree with the reviewer that increasing the E:T ratio may impact γδ T cell-mediated responses. Since the γδ T cells are effectively the targets in CD107a assays, we conducted an additional flow-based live/dead assay in co-cultures of expanded γδ T cell lines with patient-derived CLL cells, in which viability of CLL cells at various E:T ratios (1:1, 3:1, 6:1) was measured by propidium iodide uptake. Interestingly, the viability of CLL cells did decrease upon co-culture, and this decrease was more pronounced at larger E:T ratios. This decrease in viability may reflect either non-degranulating cytotoxic mechanisms, cytotoxicity mediated by γδ T cells other than Vδ1, Vδ2 and Vδ3 T cells, or may be due to the highly stressful co-culture conditions, such as nutrient deficiency, local hypoxia, lactate accumulation or the harsh conditions reflected in physically crowded co-culture conditions. CLL cells are metabolically fragile and notoriously sensitive to oxidative stress and changes in pH and nutrient availability, all of which are heavily impacted by co-culture conditions with high E:T ratios. To reflect this additional experiment, the following sections have been added to the main body of the updated manuscript:

- Lines 38 and 41-42 in the abstract
- Lines 156-157 in the updated methods
- Lines 324-333 in the updated results section
- New Figure 4
- Lines 474-489 in the updated discussion.

**Comment 4 (results):** While CD107a reflects degranulation, it does not necessarily demonstrate that target cells are being killed. Complement CD107a with direct granzyme B delivery and target death are recommended.

**Response:** We appreciate the reviewer’s suggestions and have now carried out a propidium iodide assay to measure target cell death, as described above in the response to Comment 1 from Reviewer 1.

**Comment 5 (results):** Besides the E:T ratio and complementary assays for CD107a, the negative results may also reflect (a) the need of subset purification/priming, Vδ1 T cells ranged from 13.9 to 51%, which means in some cases, Vδ1 could be too diluted to show changes. Vδ3 T cells are a minor population (1.3-25%), so CD107a negativity in this subset could be due to low numbers rather than true absence of degranulation. (b) the possibility that Vδ1/Vδ3-mediated cytotoxicity proceeds via non-degranulation pathways, which would not be captured by CD107a.

**Response: (a)** We agree with this reviewer that the negative effector functions observed may be due to a necessity to purify specific γδ T cell subsets, since it is possible that different γδ T cell subsets could regulate each other. However, we believe that the low frequencies of Vδ1 and Vδ3 T cells were not a problem, since sufficient numbers of each cell subset could be gated on using flow cytometry. We agree that γδ T cell subset purification warrants further investigation in future studies. To highlight the reviewer’s valid point, multiple allusions to this have been made in this discussion of the updated manuscript:

- Line 533-534 in the discussion states “Therefore, it may be worth selecting for and expanding Vδ1 T cell lines, rather than using polyclonal pan γδ lines.“
- Line 546-547 in the discussion states “The use of purified Vδ1 or Vδ2 T cell cultures may prove more effective in the killing of primary CLL cells.”

**(b)** We strongly agree with this reviewer that Vδ1/Vδ3-mediated cytotoxicity may proceed via non-degranulation pathways, which would not be captured by CD107a. To address this issue, further cytotoxicity assays were carried out. Firstly, a flow-based live/dead assay was conducted in co-cultures of expanded γδ T cell lines with patient-derived CLL cells, in which viability of CLL cells at various E:T ratios (1:1, 3:1, 6:1) was measured by propidium iodide uptake. The results of this experiment and the associated revisions to the manuscript are described above in Response 1 to Reviewer 1.

**Comment 6 (results):** Line 308 states PBMC from 13 CLL patients were analyzed but Table 1 indicates there are 15 patients. Please explain.

**Response:** We acknowledge the reviewer’s comment regarding the differences in sample sizes between figures. Not every patient sample used in the study (as seen in the updated version of Table 1) was used in each experiment. For instance, some samples were used in co-culture experiments, and others were used for phenotyping experiments.

**Comment 7 (results):** Figure 3F, why is sample number in ULBP3 detection different from the sample number in CD1d figure?

**Response:** As mentioned above, not every patient sample used in the study was used in each experiment. Only five out of eighteen CLL samples was used for ULBP3 phenotyping due to limited availability of a ULBP3 antibody. We believe that despite the small sample size, the trending decrease in ULBP3 still brings value in this study as a potential other mechanism of immune evasion of these cells against immune cells, and particularly γδ T cells.

**Comment 8 (results):** Figure 5 does not show ULBP3 expression after treatment in CLL donors.

**Response:** We acknowledge the reviewer’s comment that upregulation of ULBP3 by RAR-α agonists is lacking in this study. Unfortunately, limited availability of the antibody meant that this could not be carried out in CLL samples. We believe, nevertheless, that the finding that ULBP3 is significantly upregulated on healthy donor B cells is still valuable and enhances this study.

**Comment 9 (results):** Figure 6 showed healthy donor T cells alone show even higher CD107a than the positive control (PMA/ionomycin), which requires explanation.

**Response:** We acknowledge the reviewer’s comment, however statistically there is no significant difference between CD107a expression by any γδ T cell subset cultured alone compared to the positive control (PMA/ionomycin). This can be seen in the updated Figure 6 (A).

**Comment 10 (discussion):** Discussion comparing these results to previous work with iNKT-mediated killing would help interpret the negative findings.

**Response:** We strongly agree with the reviewer that reference to previous work evaluating iNKT-mediated killing would help with interpreting these negative findings. To highlight the reviewer’s valid point, allusions to this have been made in this discussion of the updated manuscript:

- Line 493-495 in the updated discussion states “Since this was not the case, and because the iNKT cell agonist glycolipid α-galactosylceramide is required to prime CLL cell for killing by iNKT cells [31], we next tested five synthetic glycolipids for their ability to induce γδ T-cell killing of untreated or ATRA-treated B cells.”
- Line 518-520 in the updated discussion states “CD1d-dependent cytotoxicity mediated by iNKT cells against CLL cells was limited to CD8+ iNKT cells, whereas their CD4+ and CD4-CD8- counterparts did not exhibit cytotoxic activity [24] and the same might be true for Vδ1 T cells.”

**Comment 11 (discussion):** Since γδ-mediated killing is not a major mechanism in CLL, providing rationale for studying this mechanism, e.g., limitations of anti-CD20 therapies and potential additive effects of alternative cytotoxic pathways, would strengthen the significance.

**Response:** We agree with the reviewer’s comments that γδ-mediated killing is not a dominant anti-CLL endogenous mechanism yet warrants investigation due to limited efficacy of anti-CD20 therapies. This has been highlighted in the introduction of the updated manuscript:

- Lines 556-559 in the updated discussion states “Despite not being a dominant endogenous anti-CLL mechanism, γδ T cell cytotoxicity warrants further investigation, given the resistance associated with anti-CD20 therapies and the advantage of being able to use γδ T cells as an allogeneic therapy”.

**Comment 12 (figures):** Gating strategy figure (Figure 1) should be moved to supplementary materials.

**Response:** The gating strategy figure (originally Figure 1) has been moved to the supplementary materials (now Figure S1).

**Comment 13 (figures):** Figure 5, please keep colors consistent with other figures.

**Response:** We agree with the reviewer and have changed the colours of bar plots to improve readability. These changes are reflected in the updated figures. In general, teal is used for healthy donor B cells and pink has been used for CLL patient PBMC. Shading within these two colours further indicates either γδ T cell subset (Figure 6A in updated manuscript), or +/- ATRA treatment (Figure 7 in updated manuscript) between healthy B cells or CLL patient PBMC. We hope that this is clearer and most intelligible for readers and thank the reviewer for their help with this.

**Comment 14 (figures):** Figure 7, please label for Vδ1/2/3 T cells in figures to improve readability.

**Response:** Vδ1/2/3 T cells have now been labelled in the updated Figure 7.

**Comment 15 (figures):** Figure legends appear misplaced.

**Response:** The figure legends/captions have been reorganised within the updated manuscript, and are placed directly after first mention in the text, in accordance with the PLOS ONE Figures guidelines.

We are grateful to our reviewers who put their time into providing these intuitive and thoughtful comments and believe the manuscript has significantly improved therewith. Should you require any additional information, please do not hesitate to contact me at your convenience.
